# Adsorption energy of oxygen molecules on graphene and two-dimensional tungsten disulfide

**DOI:** 10.1038/s41598-017-01883-1

**Published:** 2017-05-11

**Authors:** Filchito Renee Bagsican, Andrew Winchester, Sujoy Ghosh, Xiang Zhang, Lulu Ma, Minjie Wang, Hironaru Murakami, Saikat Talapatra, Robert Vajtai, Pulickel M. Ajayan, Junichiro Kono, Masayoshi Tonouchi, Iwao Kawayama

**Affiliations:** 1 Institute of Laser Engineering, Osaka University, 2-6 Yamadaoka, Suita, Osaka 565-0871 Japan; 2 Department of Physics, Southern Illinois University-Carbondale, Carbondale, Illinois 62901-4401 United States; 3 Department of Materials Science and NanoEngineering, Rice University, Houston, Texas 77005 United States; 4 Department of Electrical and Computer Engineering, Rice University, Houston, Texas 77005 United States; 5 Department of Physics and Astronomy, Rice University, Houston, Texas 77005 United States

## Abstract

Adsorption of gas molecules on the surface of atomically layered two-dimensional (2D) materials, including graphene and transition metal dichalcogenides, can significantly affect their electrical and optical properties. Therefore, a microscopic and quantitative understanding of the mechanism and dynamics of molecular adsorption and desorption has to be achieved in order to advance device applications based on these materials. However, recent theoretical calculations have yielded contradictory results, particularly on the magnitude of the adsorption energy. Here, we have experimentally determined the adsorption energy of oxygen molecules on graphene and 2D tungsten disulfide using temperature-programmed terahertz (THz) emission microscopy (TPTEM). The temperature dependence of THz emission from InP surfaces covered with 2D materials reflects the change in oxygen concentration due to thermal desorption, which we used to estimate the adsorption energy of oxygen molecules on graphene (~0.15 eV) and tungsten disulphide (~0.24 eV). Furthermore, we used TPTEM to visualize relative changes in the spatial distribution of oxygen molecules on monolayer graphene during adsorption and desorption. Our results provide much insight into the mechanism of molecular adsorption on the surface of 2D materials, while introducing TPTEM as a novel and powerful tool for molecular surface science.

## Introduction

The successful isolation of monolayer graphene in 2004 and its remarkable properties found subsequently have paved the way for a new research field of two-dimensional (2D) atomic layer materials^1–4^. Many other 2D materials have since been discovered with a wide range of characteristics, from metallic to semiconducting to insulating, opening up exciting new opportunities for the development of devices based on monolayers, bilayers, and heterostructures of 2D materials^5–8^. However, since these materials typically consist of one or a few atomic layers, their properties are extremely susceptible to perturbations from their environment. Exposure to gases, for example, has been shown to drastically affect their electrical and optical properties^9–15^, which means that in order to realize 2D-materials-based devices, it is crucial to understand and control the influence of gas adsorption and desorption dynamics on their properties.

Of the possible gas adsorbates/contaminants, oxygen (O_2_) is one of the most important because not only it significantly alters the properties through doping, it is also the second most abundant gas in the atmosphere and is therefore highly likely to affect the performance of devices in practical applications. Though theoretical simulations proved to be useful in understanding the interaction of O_2_ molecules and/or O atoms with 2D materials, conflicting results for the adsorption energies were obtained due to the inability of the approximation functionals used to properly describe the dispersion forces^16–23^. Knowing the correct value of the adsorption energy is important since it describes the strength of the interaction of the adsorbate with the 2D material, as well as the extent to which its properties are altered. Experimental measurements using thermal desorption spectroscopy (TDS) [also known as temperature-programmed desorption (TPD)] were done as a complementary investigation to the theoretical results^24–26^. However, these measurements were done only on highly-oriented pyrolytic graphite (HOPG), which is the bulk form of graphene. Another drawback is that in TDS/TPD, the desorption of molecules is usually measured from a large sample area, failing to provide information on local adsorption and desorption dynamics. This is a significant disadvantage because the large surface-to-volume ratio enhances the importance of surface interactions in 2D materials.

Here, we used temperature-programmed terahertz (THz) emission microscopy (TPTEM) to probe local O_2_ adsorption and desorption dynamics in graphene and tungsten disulfide (WS_2_) with micrometer resolution. THz spectroscopy has been proven to be a powerful tool in studying the chemical and physical processes in a variety of systems^27, 28^. In our work, we used the THz emission from the surface of InP coated with monolayer graphene to observe the relative concentration of O_2_ adsorbates^29, 30^. In this non-contact and non-destructive method, femtosecond infrared (IR) laser pulses are used to generate THz waves from a spot on the substrate surface. Absorption of both the incident IR and the generated THz radiation by graphene (and other 2D materials) is very small compared to the effects of adsorption of O_2_ molecules on the 2D materials to the THz emission, which allows us to use THz emission as a probe in studying O_2_ adsorption.

The waveform of the THz radiation changes dramatically when O_2_ adsorbates are present on the graphene surface because the electric dipoles induced by the adsorbates modify the surface depletion field in InP, which changes both the polarity and magnitude of the emitted THz wave. By correlating the changes in the waveform of THz emission and the relative concentration of O_2_ molecules on the sample surface as a function of temperature, we were able to obtain the first experimental value of the adsorption energy of locally physisorbed O_2_ molecules on graphene and WS_2_. Furthermore, the method can be used to directly visualize the relative amount of O_2_ molecules on the sample surface, allowing us to monitor the spatial distribution of O_2_ adsorbates in monolayer graphene under controlled conditions. This capability allowed us to observed the influence of sample surface non-uniformity on O_2_ adsorption and desorption, as well as the reported UV-illumination-induced enhancement of O_2_ adsorption^31–33^.

## Results and Discussion

### THz emission from Graphene/InP surface

**Figure 1:**
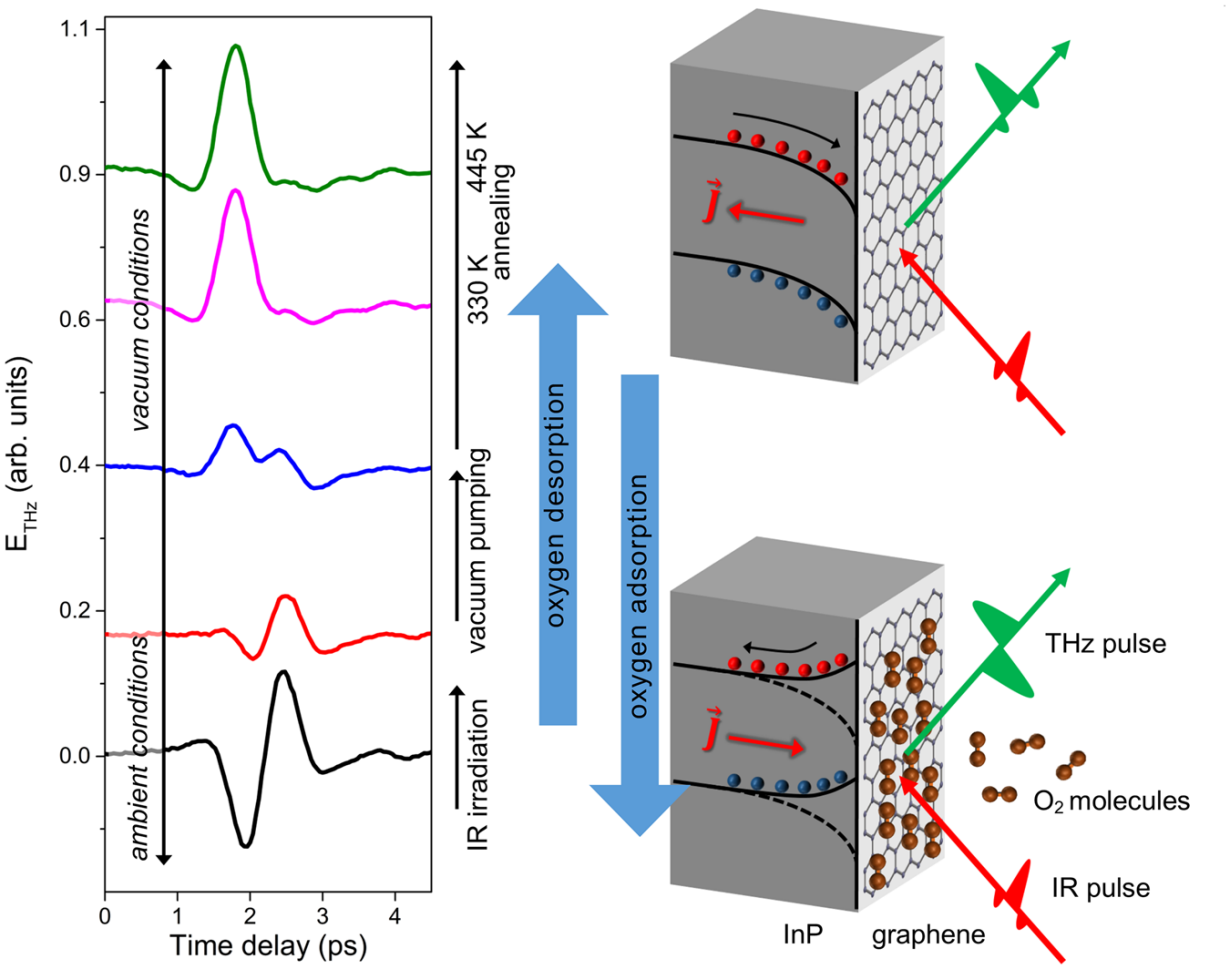
THz emission waveforms from Graphene/InP measured by TPTEM. Initially, when O_2_ molecules are adsorbed in graphene, modification in the surface depletion field causes the photoexcited current to move towards the surface of InP, generating THz radiation with waveform containing a dip at ~2 ps and a peak at ~2.5 ps. Desorption of O_2_ by IR irradiation and/or vacuum pumping causes both features to gradually vanish, followed by the appearance of a positive peak at ~2 ps. Subsequent annealing removes more adsorbates, and the THz emission from graphene/InP becomes very similar to the emission from bare InP, signifying that the photoexcited current flows towards the substrate. Exposure of sample to air allows O_2_ to be adsorbed on graphene, which reverts the THz emission back to the initially observed waveform.

The time-domain waveforms of the THz emission from graphene/InP changes with the removal of O_2_ adsorbates from the graphene surface as shown in Fig. 1. Initially, when adsorbates are present on the graphene surface, the transient current excited by femtosecond IR pulses flows towards the surface of InP, generating a THz waveform with two peaks of opposite polarities – a dip at ~2 ps and a peak at ~2.5 ps. Significant O_2_ desorption occurs upon vacuum pumping, and the change in the THz waveforms reflects the influence of the decrease in adsorbate concentration on the photoexcited current in InP. Both features in the THz waveform gradually vanish, followed by the appearance of a positive peak at ~2 ps. Irradiation with femtosecond IR pulses also promotes desorption of O_2_, with the rate of desorption dependent on the laser fluence^29^. With further removal of adsorbates by annealing, the photoexcited current flows towards the bulk, and the THz emission from graphene/InP becomes similar to that of the bare InP substrate. O_2_ adsorption by exposure to air reverts the THz waveform back to the initially observed waveform, and this adsorption is dramatically enhanced under UV light illumination. The change in the THz emission from graphene/InP (due to O_2_ adsorption/desorption) shown in Fig. 1 can be semi-quantitatively modeled using the relation ${\vec{E}}_{T}=x{\vec{E}}_{1}+(1-x){\vec{E}}_{2}$, where ${\vec{E}}_{1}$ and ${\vec{E}}_{2}$ are the initial (black) and the final (green) waveforms in Fig. 1, respectively, and *x* (0 ≥ *x* ≥ 1) is their relative weight which is proportional to the concentration of adsorbed O_2_ on graphene^29^.

### THz emission from 2D-coated InP at different conditions

**Figure 2:**
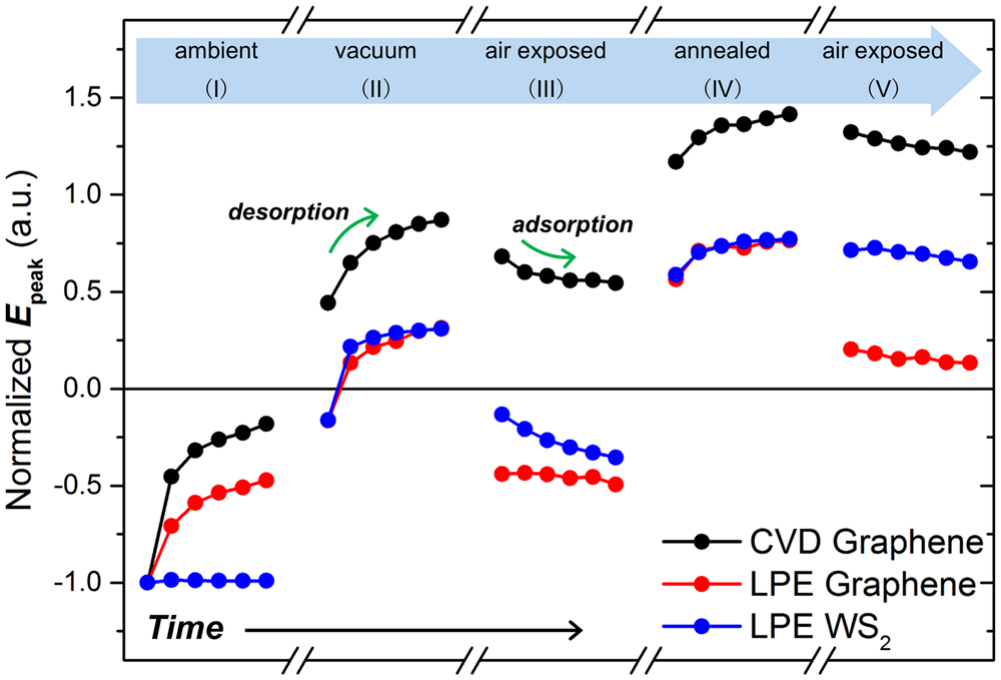
Temporal evolution of the THz peak amplitude at different conditions. The peak values (first peak at ~2 ps) are normalized with respect to the first peak of the initial THz emission measured in ambient conditions (I). The THz emission is recorded at 2-minute intervals after changing the environmental condition. Increase (decrease) in the peak value means net desorption (adsorption).

With the TPTEM setup, we monitored the THz emission from InP coated with graphene prepared by chemical vapor deposition (CVD) and liquid phase exfoliation (LPE), as well as with WS_2_ nanosheets prepared by LPE. This is done to determine how sensitive is the change in the THz emission from 2D-coated InP due to O_2_ adsorption/desorption, and if TPTEM can be used to observe changes in adsorption/desorption dynamics at different conditions. Differences in the temporal evolution of the THz emission peak (at ~2 ps) from the samples can be observed under continuous IR irradiation at various conditions as shown in Fig. 2 (see Figs S1–S3 in the Supporting Information for the waveforms and a more detailed discussion). These differences in the peak variation is most likely related to differences in the affinity of samples to O_2_ molecules; for instance, 2D materials prepared by LPE usually contain more structural defects than those prepared by CVD, and these defects can act as adsorption sites for molecules^23, 34–36^.

### Estimation of adsorption energy using TPTEM

We then used TPTEM to measure the adsorption energy of O_2_ in graphene and WS_2_. The experiment was designed to be similar to TDS/TPD, wherein the sample of interest is pre-exposed to a gas adsorbate and then heated in a controlled fashion while monitoring the partial pressures of the evolved molecular species by using, for example, a mass spectrometer^37, 38^. The main difference of TPTEM from TDS/TPD is that it can be used to monitor the relative concentration of the remaining adsorbates within the spot on the sample illuminated by the femtosecond laser. This means that it is possible to probe not only desorption dynamics but also adsorption dynamics in 2D materials within the excitation spot.

**Figure 3:**
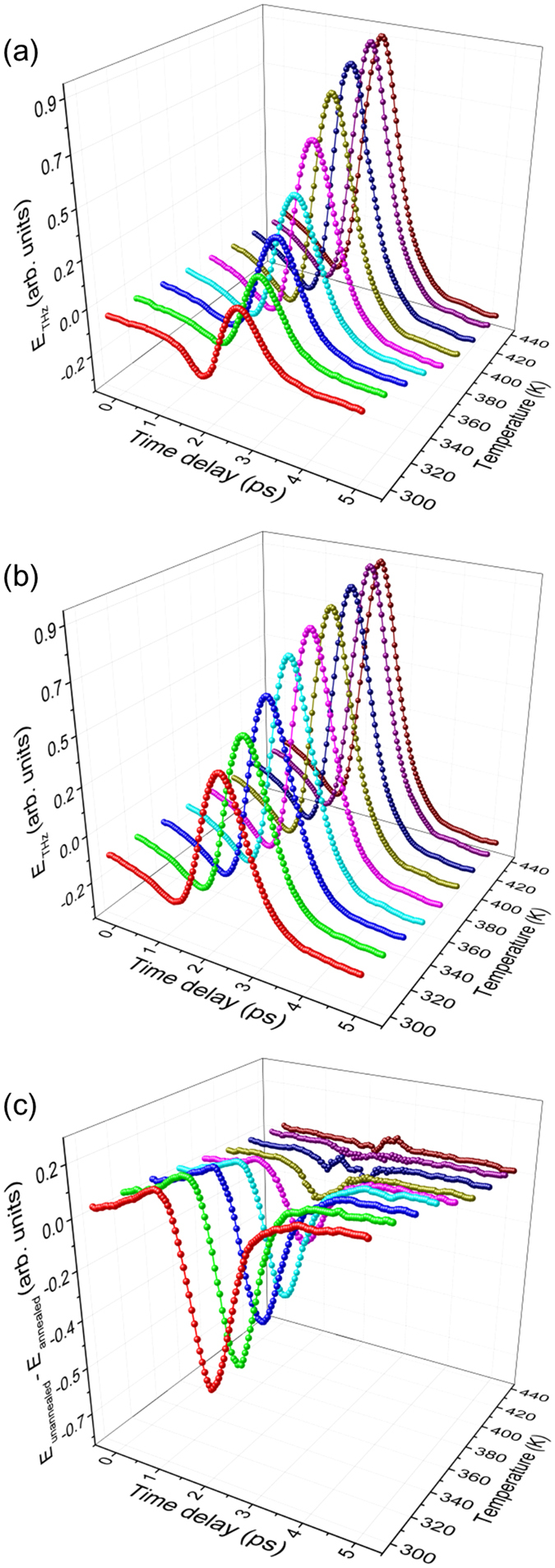
THz emission from CVD Graphene/InP at different temperatures in vacuum (1 × 10^−2^Pa). (**a**) Pre-exposed in air, and (**b**) after annealing at 445 K for 1 hour in vacuum. (**c**) ${{\boldsymbol{E}}}_{{\bf{u}}{\bf{n}}{\bf{a}}{\bf{n}}{\bf{n}}{\bf{e}}{\bf{a}}{\bf{l}}{\bf{e}}{\bf{d}}}-{{\boldsymbol{E}}}_{{\bf{a}}{\bf{n}}{\bf{n}}{\bf{e}}{\bf{a}}{\bf{l}}{\bf{e}}{\bf{d}}}\cong \,{{\boldsymbol{E}}}_{{{\bf{O}}}_{2}}$ at different temperatures for CVD graphene/InP.

**Figure 4:**
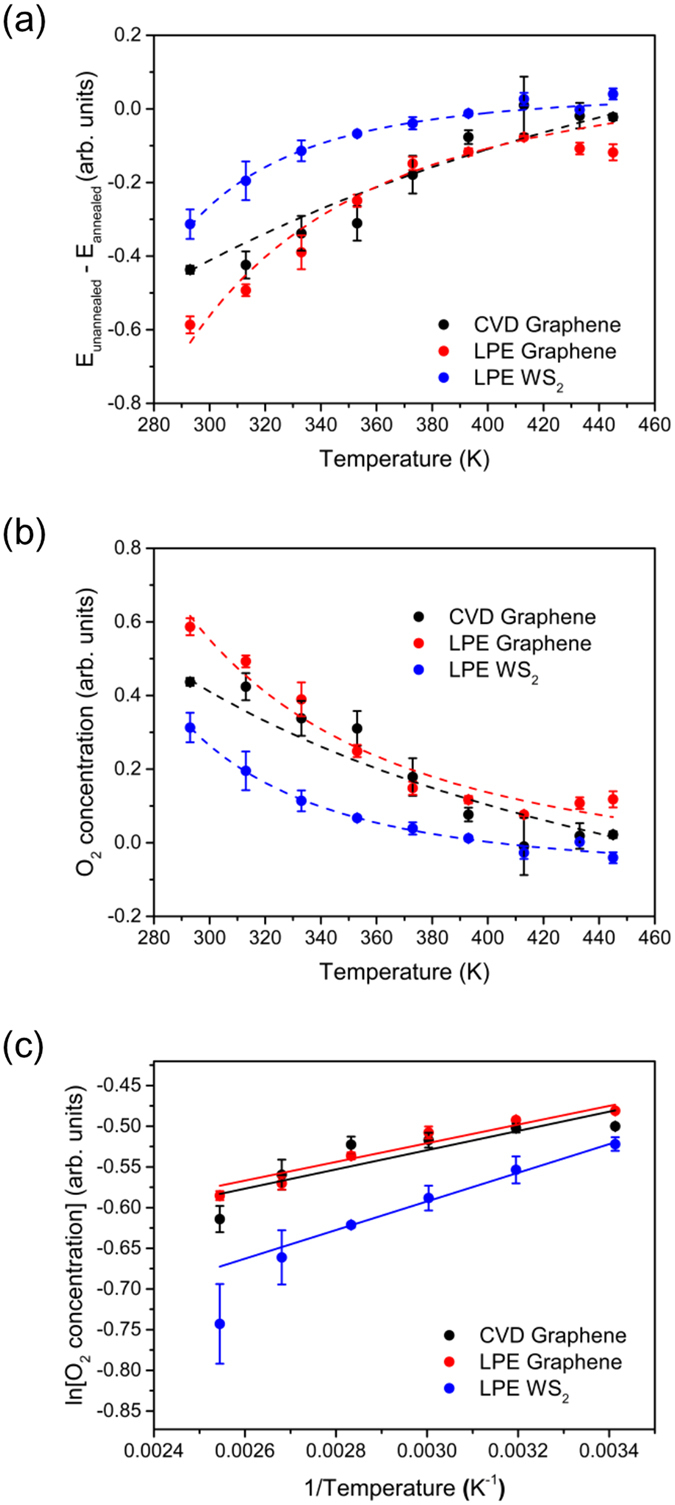
Determination of the desorption energy. (**a**) Peak versus temperature of waveforms (${{\boldsymbol{E}}}_{{\bf{u}}{\bf{n}}{\bf{a}}{\bf{n}}{\bf{n}}{\bf{e}}{\bf{a}}{\bf{l}}{\bf{e}}{\bf{d}}}-{{\boldsymbol{E}}}_{{\bf{a}}{\bf{n}}{\bf{n}}{\bf{e}}{\bf{a}}{\bf{l}}{\bf{e}}{\bf{d}}}\cong \,{{\boldsymbol{E}}}_{{{\bf{O}}}_{2}}$) for all samples. (**b**) Concentration (***N***_***ad***_) of O_2_ adsorbates on surface of samples as a function of temperature. (**c**) **In**(***N***_***ad***_) versus $1/{\boldsymbol{T}}$ with linear fit for calculating desorption energy.

The temperature dependence of THz radiation from CVD graphene/InP before and after annealing at 445 K for 1 hour are shown in Fig. 3a and b, respectively (see Supporting Information for an explanation of the behavior of THz emission as a function of temperature, and for the temperature dependence of THz emission from LPE graphene/InP, and LPE WS_2_/InP in Fig. S4a–S4d). The amount of O_2_ adsorbates on the surface decreases with increasing temperature, and we can separate its effect on the measured THz waveforms by simply subtracting the THz emission of annealed samples from the unannealed samples, or ${E}_{{\rm{unannealed}}}-{E}_{{\rm{annealed}}}\cong \,{E}_{{{\rm{O}}}_{2}}$. The samples were annealed at 445 K for 1 hour in vacuum to make sure that the *E*_annealed_ waveforms represent emission from samples without adsorbates. Annealing at this temperature under vacuum conditions should completely remove physically adsorbed O_2_ molecules from the surface of monolayer graphene based on optical conductivity measurements (see Fig. S5 in the Supporting Information). Figure 3c shows the resulting waveforms for CVD graphene/InP (see Fig. S4e and S4f in Supporting Information for the waveforms for LPE graphene/InP and LPE WS_2_/InP, respectively), while in Fig. 4a we plot the change in peak values for all samples, showing decrease in absolute value with increasing temperature. The adsorbates were completely removed for CVD graphene around 413 K. However, for LPE graphene, longer annealing at 445 K was needed for further desorption, implying that there are more strongly bonded adsorbates in LPE graphene. For LPE WS_2_, the absolute peak value reaching zero at 413 K could mean either (a) complete O_2_ desorption at this temperature, or (b) no further desorption happened during the annealing from 413 K to 445 K. Considering the relatively larger adsorption energy in WS_2_ and the high possibility of the existence of chemisorbed species^21, 22^, the latter is more likely the case.

THz emission from InP primarily occurs through the surge current effect^39, 40^. In the far-field approximation, the amplitude of this radiation is proportional to the time derivative of the surge current, given by^41^,

1 $${E}_{THz}\propto \frac{\partial J(t)}{\partial t}.$$

The surge current, *J*(*t*), is due to the transient photocarriers generated by IR excitation. One of the simplest approximations of this surge current (density) is given by the expression^42^,

2 $$J(t)=q\mu (t)n(t){E}_{d}(t),$$

where *q* is the electron/hole charge, *μ*(*t*) is the electron/hole mobility, *n*(*t*) is the carrier density, and *E*_*d*_(*t*) is the driving electric field, which in this case is the built-in surface field in InP. The parameters *μ*(*t*), *n*(*t*), and *E*_*d*_(*t*) determine the observed THz field through equation (1).

Our experimental configuration as well as the observed results lead us to believe that during the O_2_ adsorption/desorption process, the change in the THz radiation from graphene/InP is mainly due to the change in *E*_*d*_(*t*) (see Supporting Information for more details), which in turn is caused by the electric field on the surface of InP due to the dipoles induced by the O_2_ adsorbates in graphene (*E*_*dipole*_)^29^. Simulations show that charge transfer occurs between the O_2_ molecules and carbon atoms in graphene^19^, and similarly for other 2D materials^11, 22^, supporting the possibility of the existence of these electric dipoles. If we denote the modified driving field due to the presence of adsorbates as *E*_*d*,2_ = *E*_*d*,1_ + *E*_*dipole*_, then the change in the THz emission from graphene/InP due to O_2_ adsorbates can be obtained using equations (1) and (2) as,

3 $${E}_{THz,2}-{E}_{THz,1}\propto \frac{\partial {J}_{2}}{\partial t}-\frac{\partial {J}_{1}}{\partial t}\propto {E}_{d,2}-{E}_{d,1}={E}_{dipole},$$

where the subscripts 2 and 1 represent the state with (unannealed) and without (annealed at 445 K) adsorbed O_2_, respectively. *E*_*dipole*_ is directly proportional to the amount of charge comprising the dipole, which means that the peak of the waveforms plotted in Fig. 4a is directly proportional to the concentration of the O_2_ adsorbates on the surface.

The adsorbate concentration is plotted as a function of temperature in Fig. 4b for all samples. From these data, we can then determine the activation energy of desorption (amount of energy needed to remove the adsorbate from the surface) through the Polanyi-Wigner equation (PWE)

4 $$-\frac{d{N}_{ad}}{dt}=v{N}_{ad}^{x}\exp (-\frac{{E}_{des}}{RT}),$$

where $-\frac{d{N}_{ad}}{dt}$ is the desorption rate, *v* is the attempt frequency, *N*_*ad*_ is the instantaneous adsorbate concentration, *x* is the order of desorption, *E*_*des*_ is the activation energy of desorption, *R* is the gas constant, and *T* is the temperature^37^. Solving equation (4) for ${N}_{ad}^{x}$ and taking the natural logarithm of both sides, we obtain

5 $$\mathrm{ln}({N}_{ad}^{x})=\,\mathrm{ln}(\frac{-d{N}_{ad}/dt}{v})+\frac{{E}_{des}}{R}(\frac{1}{T}),$$

which is a linear relationship between of $\mathrm{ln}({N}_{ad}^{x})$ and versus 1/*T* with a slope of *E*_*des*_/*R*.

Plots of $\mathrm{ln}({N}_{ad}^{x})$ versus 1/*T* (with *x* = 1) with a linear fit for each sample are shown in Fig. 4c. We assumed a first-order (*x* = 1) or non-dissociative desorption in our data analyses since a large energy barrier would have to be overcome for O_2_ molecules to dissociate into O atoms once adsorbed in graphene and MoS_2_ (a similar material to WS_2_)^11, 19^. In this case, the activation energy of desorption obtained is approximately equal to the adsorption energy^37^. We then calculated *E*_*des*_ from the slope of the linear fits in Fig. 4c, and these values are tabulated in Table 1, together with values found in the literature for comparison (values were converted to eV for easier comparison). The adsorption energies obtained using TPTEM are similar to reported theoretical and experimental values of the adsorption energy for physisorbed O_2_ molecules on WS_2_^22^ and on graphene and HOPG^19, 20, 24–26^. The chemistry and morphology of the supporting substrate significantly influence molecular interaction in graphene^43, 44^ and possibly in other 2D materials as well. This influence becomes weaker with increasing number of layers^45–47^. In our work, similar adsorption energy values were obtained for CVD monolayer graphene and LPE graphene multilayer (typically 3–4 layers) nanosheets. This leads us to believe that these values are good approximation of the actual adsorption energy of oxygen molecules on pristine graphene.

**Table 1:** Measured adsorption energy for physisorbed O_2_ on graphene and tungsten disulfide using temperature-programmed THz emission microscope.

Sample	Adsorption energy (eV)	
	This experiment (TPTEM)	Literature
Graphene	0.16^*^, 0.15^**^	0.13^*a*^, 0.15^*b*^, 0.124^*c*^, 0.04^*d*^, 0.1^*e*^, 0.01^*f*^
Tungsten disulfide	0.24^**^	0.213^*g*^

*Chemical vapor deposition-grown sample. ^**^Liquid phase exfoliated sample. ^*a*^O_2_ molecule physisorbed on undoped graphene, obtained by first-principles calculations^19^. ^*b*^O_2_ molecule physisorbed on pristine graphene, obtained by first-principles calculations^20^. ^*c*^(12.0 kJ/mol) O_2_ molecule physisorbed on highly oriented pyrolytic graphite, obtained by thermal-desorption spectroscopy^24–26^. ^*d*^O_2_ molecule physisorbed on single layer graphite, obtained by density-functional calculations^17^. ^*e,f*^O_2_ molecule physisorbed on graphene, obtained by spin-unrestricted density-functional calculations^18^. ^*g*^O_2_ molecule physisorbed on WS_2_ monolayer (on the most favorable site of adsorption), obtained by first-principles calculations^22^.

Only the physisorbed O_2_ molecules were removed in the temperature range considered in measuring *E*_*des*_. However, it is clear that for LPE graphene a significant number of adsorbates remain at *T* > 400 K, which represent the O_2_ molecules (or O atoms) attached to graphene at higher adsorption energies. The inherent defect sites in LPE graphene could lead to spontaneous chemisorption of oxygen, and increasing the temperature range in this experiment should allow us to probe the desorption dynamics of these chemisorbed species.

### Spatial distribution of O_2_ molecules in monolayer graphene

The adsorption energies presented in Table 1 are basically averaged over the probed surface area, which is roughly the area illuminated by the femtosecond excitation pulses (with a diameter of approximately 1.35 mm). This area is still quite large in the context of 2D materials. To better understand the O_2_ adsorption/desorption dynamics at different sites in the sample surface, we adjusted the excitation spot size to ~0.2 mm and used the imaging capability of TPTEM to study the spatial distribution of adsorbed O_2_ molecules in CVD graphene at various controlled conditions. This can be done by placing the sample chamber on a computer-controlled stage. First, we checked the distribution of adsorbed O_2_ molecules within a ~1.65 × 1.65 mm^2^ surface area of the monolayer graphene deposited on InP, after exposing the sample in air for a long time. The acquired image (Fig. S6a in Supporting Information) clearly shows differences in THz amplitude in the scanned area, implying an inhomogeneous distribution of O_2_ molecules. This inhomogeneity is most likely caused by non-uniformity of the sample surface (*e.g*., presence of structural defects and/or unwanted doping during sample preparation). Structural defects and doping in graphene have significant effects on its reactivity towards adsorbates^19, 20, 23, 34, 35, 48^.

**Figure 5:**
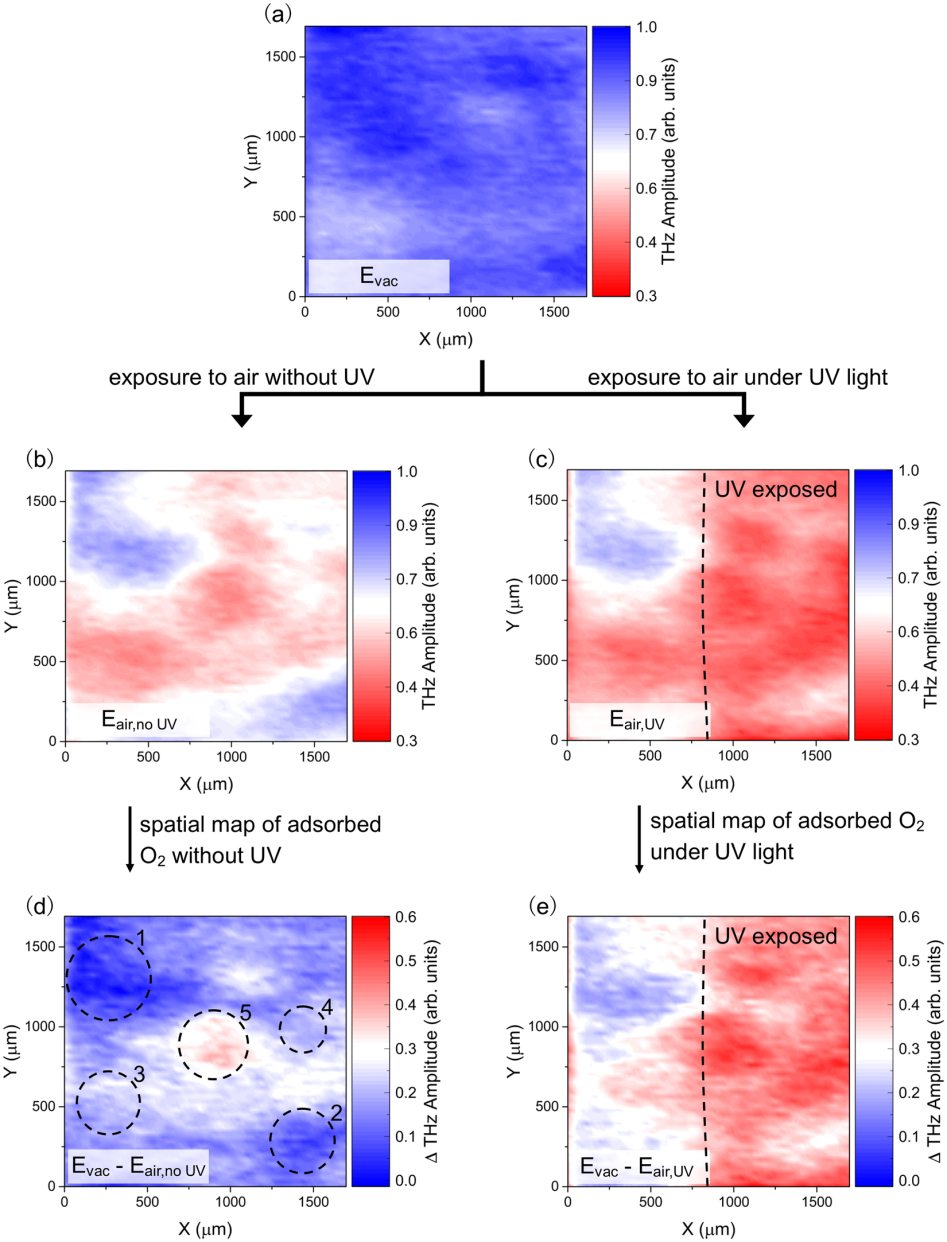
THz amplitude mapping during the O_2_ adsorption process. (**a**) THz amplitude mapping for CVD graphene/InP after annealing at 445 K in vacuum. THz mapping of the same area in sample after exposure to air for a few minutes (**b**) without and (**c**) with UV illumination. The difference in THz mapping images before and after exposure to air gives a visualization of adsorbed O_2_ molecules in the graphene surface. (**d**) Spatial map of adsorbed O_2_ molecules in graphene after exposure to air without UV illumination which reveals regions with different “natural” affinities to O_2_ molecules. Regions 1 and 2 (5) show the least (highest) amount of adsorbates after exposure to air, whereas regions 3 and 4 are areas with a fair amount of adsorbates. (**e**) Spatial map of adsorbed O_2_ molecules after exposure to air under UV illumination, showing significantly higher concentration of O_2_ adsorbates in the UV exposed part. In the THz amplitude maps (**a**–**c**), the blue (red) end of the scale signifies less (more) O_2_ molecules on the graphene surface, while in the ΔTHz amplitude maps (**d** and **e**), the blue (red) end of the scale signifies less (more) O_2_ molecules adsorbed/added on graphene during exposure to air.

The non-uniformity of the surface and its influence on adsorption/desorption dynamics can be clearly seen by taking THz emission images under controlled conditions. First, we removed the adsorbates in the sample by annealing it at 445 K in vacuum for at least 5 minutes, and then we monitored the spatial distribution of the relative amount of adsorbed O_2_ molecules after exposure to air for a few minutes (see Fig. S7a for changes in the THz waveforms). Figure 5a shows the THz amplitude map taken after removal of adsorbates from the sample surface. We then mapped the change in the THz amplitude of the same area after exposing the sample in air for a few minutes without (Fig. 5b) and with (Fig. 5c) UV illumination, which has been shown to enhance the adsorption of O_2_ in graphene^31–33^. The difference between the THz images before (vacuum condition) and after air exposure is taken as the measure of the adsorbed O_2_ molecules. Figure 5d is the spatial distribution of O_2_ adsorbates after exposure to air, which shows the “natural” affinity to O_2_ molecules, *i.e*., O_2_ adsorption process without external influence like UV light, of different regions in the sample surface. Regions 1 and 2 (5) in Fig. 5d show the least (highest) amount of adsorbates after exposure to air, whereas regions 3 and 4 are areas with a fair amount of adsorbates. Interestingly, the identified regions in Fig. 5d also showed different behaviors during the desorption process (see Figs S8–S9 in the Supporting Information). Illumination with UV during exposure to air promotes more O_2_ adsorption, and this dramatic effect can be seen in Fig. 5e, where UV light was selectively focused on the graphene surface during exposure to air. The image clearly shows a higher concentration of adsorbates in the area exposed to UV.

## Conclusion

In conclusion, we have developed the temperature-programmed THz emission microscope (TPTEM) system and studied the adsorption and desorption dynamics of O_2_ molecules in graphene and WS_2_ with micrometer spatial resolution. Thermal annealing at 445 K shows complete removal of the physisorbed O_2_ molecules in graphene. We estimate the adsorption energy of O_2_ molecules physisorbed on graphene and WS_2_ to be ~0.15 eV and ~0.24 eV, respectively. Spatial mapping of adsorbed O_2_ molecules in monolayer graphene under controlled conditions also reveals the effects of sample non-uniformity on O_2_ adsorption and desorption dynamics. The regions in graphene which are identified to have a higher affinity to O_2_ (and thus had more adsorbed molecules upon exposure to air) also indicate a lower adsorption energy between the O_2_ molecules and the graphene surface. In addition, we show that the imaging capability of TPTEM combined with its feasibility in doing quantitative measurements makes it a valuable tool in studying O_2_ adsorption and desorption dynamics in 2D materials in a non-destructive manner. Since the primary requirement for this technique to work is that the gas adsorbates should induce local electric dipoles that will change the THz emission from InP, TPTEM is expected to be useful for studying the adsorption and desorption dynamics of other types of gas molecules in any 2D material, such as NH_3_, as long as charge transfer occurs between the adsorbate and the 2D material in question. All these results promise the possibility of doing local spatiotemporal studies on the molecular adsorption and desorption on a variety of 2D materials.

## Materials and Methods

### Sample Fabrication

Using a chemical vapor deposition (CVD) technique, methane was used as precursor to synthesize monolayer graphene on a copper foil^49^. Thin poly(methyl methacrylate) (PMMA) film was deposited on top of graphene to act as supporting layer during the wet transfer method. Diluted ammonium persulfate solution was used to dissolve the copper foil and then washed several times in deionized water. The floating PMMA/graphene film was carefully transferred onto InP (100) substrate to produce the CVD graphene/InP sample and air-dried before removing the PMMA film using acetone. Bulk graphite and WS_2_ (Sigma Aldrich) were used as starting materials to produce nanosheets using the liquid phase exfoliation (LPE) technique as previously described^50^. After annealing, each powder sample is dispersed in isopropyl alcohol and then exfoliated by applying ultrasound using a horn-tip sonicator, followed by centrifugation and decantation of the supernatant to produce the final nanosheets dispersions. 2D materials produced this way are typically few-layered nanosheets with varying laterial dimensions. The dispersions were then drop-casted onto mildly heated semi-insulating InP (100) substrate to make the LPE graphene/InP and LPE WS_2_/InP samples.

### Temperature-Programmed THz Emission Microscopy

The samples were optically excited using near-infrared pulses with a duration of ~100 fs and photon energy of 1.55 eV from a Ti:sapphire laser. The excitation beam was incident on the sample surface at an angle of 45°. The beam diameter was adjusted by changing the relative distance between the sample and the focusing lens nearest to it. In the experiments for determining the adsorption energy, the excitation beam power used was 30 mW and the beam diameter was ~1.35 mm, while in the imaging measurements, the power was 20 mW and the diameter was ~0.2 mm. In all experiments, the laser fluence was a few orders of magnitude lower than the damage threshold reported for graphene^51, 52^. At this range of laser fluence, THz radiation is generated mainly by ultrafast surface surge-currents in the semi-insulating InP substrates^39, 40^. THz radiation emitted by samples were focused onto a dipole-shaped low-temperature-grown GaAs photoconductive switch using a pair of off-axis parabolic mirrors. The output current from the switch was fed into a lock-in amplifier which was referenced to a 2.0 kHz optical chopper signal. During the measurements, the samples were placed in a vacuum chamber equipped with a PID temperature controller. The vacuum chamber containing the sample was mounted on a computer-controlled sample stage for THz imaging.

## Electronic supplementary material

Adsorption energy of oxygen molecules on graphene and two-dimensional tungsten disulfide
