# Supplementary material for: Adsorption energy of oxygen molecules on graphene and two-dimensional tungsten disulfide
Source: Sci Rep. 2017 May 11;7:1774. doi: 10.1038/s41598-017-01883-1 (PMC5431967; doi:10.1038/s41598-017-01883-1)
Supplement: Supplementary file 1 — Adsorption energy of oxygen molecules on graphene and two-dimensional tungsten disulfide [file 41598_2017_1883_MOESM1_ESM.pdf]

## Supporting Information

# Adsorption energy of oxygen molecules on graphene and two-dimensional tungsten disulfide

*Filchito Renee Bagsican*[†], *Andrew Winchester*[‡], *Sujoy Ghosh*[‡], *Xiang Zhang*[§], *Lulu Ma*[§], *Minjie Wang*[§ ‖], *Hironaru Murakami*[†], *Saikat Talapatra*[‡], *Robert Vajtai*[§], *Pulickel M. Ajayan*[§], *Junichiro Kono*[§ ‖ ※ †], *Masayoshi Tonouchi*[†], *and Iwao Kawayama*[†]

[†] Institute of Laser Engineering, Osaka University, 2-6 Yamadaoka, Suita, Osaka 565-0871, Japan
[‡] Department of Physics, Southern Illinois University-Carbondale, Carbondale, Illinois 62901-4401, United States
[§] Department of Materials Science and NanoEngineering, [‖] Department of Electrical and Computer Engineering, and [※]Department of Physics and Astronomy, Rice University, Houston, Texas 77005, United States

Initial measurements were done to determine how sensitive is the change in the THz emission from InP coated with graphene due to $O_2$ adsorption/desorption, and if TPTEM can be used to observe changes in adsorption/desorption dynamics at different conditions. Figures S1-S3 show the temporal evolution of the THz waveforms from CVD graphene/InP, LPE graphene/InP, and LPE $WS_2$/InP, respectively, under continuous irradiation with femtosecond IR pulses at a fluence of $4.42 \times 10^{-7}$ J/cm$^2$. The measurements were taken every 2 minutes at different experimental conditions. Firstly, (a) the samples were pre-exposed in air for a long time, then the changes in the THz emission under IR irradiation were monitored in ambient conditions. Next in (b), the measurements were taken after keeping the samples in vacuum ($1 \times 10^{-3}$ Pa) overnight and after immediately exposing them in atmospheric air. Then at (c), the same measurements as in (b) were also done but this time the samples were annealed at 400 K for 1.5 hours in vacuum. Figure 2 in the main text shows the variation with time in the amplitude (first peak at ~2 ps) of the THz waveforms, with values normalized with respect to the first peak of initial THz waveform measured at ambient conditions. Changes in the THz amplitude (and polarity) show the relative changes in the concentration of $O_2$ adsorbates. In the data presented in Fig. 2, a relative increase in the peak amplitude indicates a net desorption while a relative decrease indicates a net adsorption. Several differences in the behavior of the change in the peak amplitudes of the THz emissions (i.e., change in relative concentration of adsorbed $O_2$) from samples are noticeable, most likely due to the differences in the chemical nature of the 2D material and to the surface morphology or quality

of the samples. 2D materials prepared by LPE usually contain more defects than those prepared by CVD, and these defects can act as adsorption sites for molecules. Initially, irradiation with femtosecond laser pulses causes removal of adsorbates in both CVD graphene and LPE graphene, though faster desorption is observed for CVD graphene (Fig. 2, I). For LPE $WS_2$ (Fig. 2, I), no change in the THz emission was observed at ambient conditions, i.e., no net desorption/adsorption. After pumping the vacuum chamber overnight, significant desorption was observed in all samples (Fig. 2, II). However, the negative polarity of initial THz waveforms from both LPE graphene and LPE $WS_2$ indicate that there are still significant $O_2$ adsorbed on them compared to CVD graphene even after pumping out air from the chamber. Continuous irradiation with laser pulses caused further desorption of $O_2$ from samples. After breaking the vacuum condition, net adsorption occurs even when the samples were continuously irradiated with the femtosecond laser (Fig. 2, III). For both LPE samples, the polarity of THz waveform is reversed almost immediately after exposure to air, indicating faster adsorption rate compared to CVD graphene. The peak amplitude for LPE graphene did not change significantly after measuring the first waveform (already saturated). After annealing the samples at 400 K for 1.5 hours, substantial $O_2$ adsorbates were removed from the surface (Fig. 2, IV). However, the annealing process did not completely remove the $O_2$ adsorbates because we can still observe some desorption caused by femtosecond laser irradiation. After breaking the vacuum and exposing the samples to air, adsorption was observed for all samples but the amount of adsorbed $O_2$ is significantly lower compared to the unannealed case (Fig. 2, V). Removing moisture from samples by thermal annealing significantly hindered adsorption for CVD graphene and LPE $WS_2$. Previous experiments show that graphene and $WS_2$ that have not undergone thermal annealing to remove moisture can be easily oxidized by exposure to UV.[1,2] Interestingly, adsorption in LPE graphene was higher compared to CVD graphene and LPE $WS_2$ even after annealing. Previous reports have shown that introduction of defects increase the interaction of gases with graphene which explains the observed significant $O_2$ adsorption in LPE graphene.[3–6] For LPE $WS_2$ however, the removal of moisture significantly hindered $O_2$ adsorption even on the defect sites. It seems that the presence of water molecules is needed for $O_2$ molecules to be adsorbed in $WS_2$, and that they attach more strongly once adsorbed as implied by the measurements in Fig. S3a.

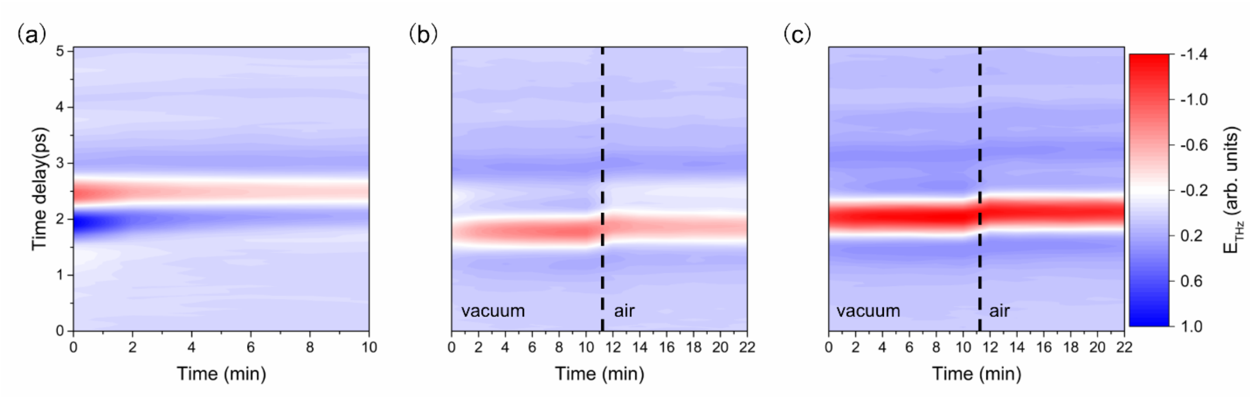


**Figure S1**. Temporal evolution of THz emission from CVD graphene/InP with continuous irradiation of femtosecond IR pulses at a fluence of $4.42\times10^{-7}$ J/cm$^2$ at different conditions: (a) ambient conditions, pre-exposed sample, (b) vacuum ($1\times10^{-3}$ Pa) overnight, and then exposed in air, and (c) same measurement in (b) but with sample annealed at 400 K for 1.5 hours in vacuum.

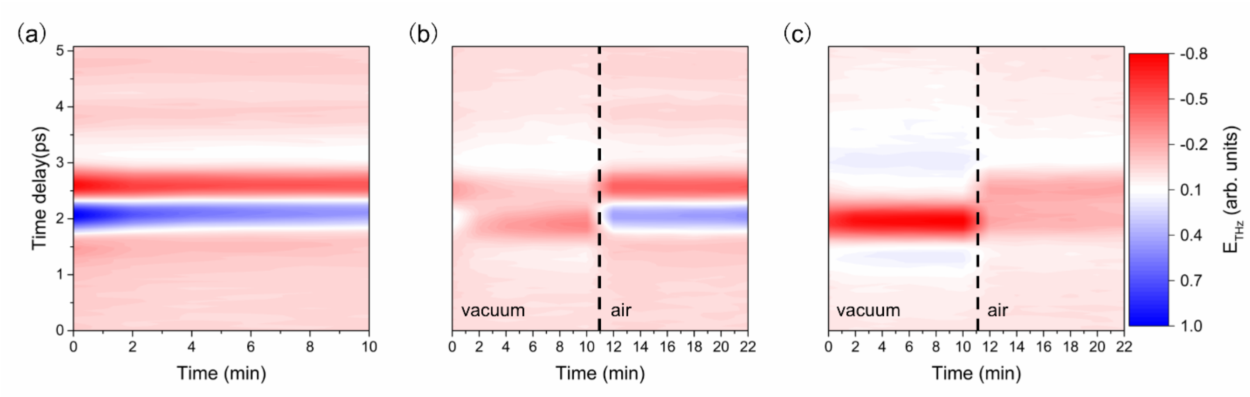


**Figure S2**. Temporal evolution of THz emission from LPE graphene/InP with continuous irradiation of femtosecond IR pulses at a fluence of $4.42\times10^{-7}$ J/cm$^2$ at different conditions: (a) ambient conditions, pre-exposed sample, (b) vacuum ($1\times10^{-3}$ Pa) overnight, and then exposed in air, and (c) same measurement in (b) but with sample annealed at 400 K for 1.5 hours in vacuum.

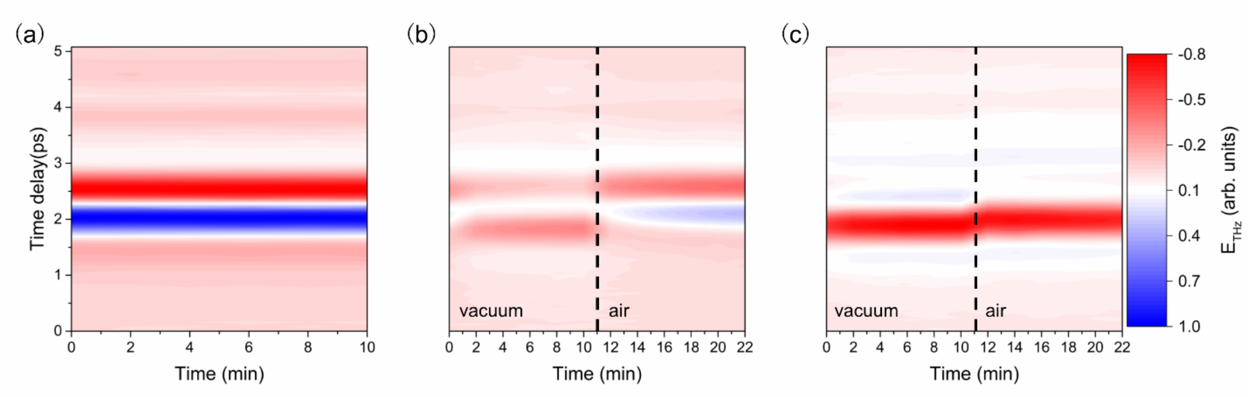


**Figure S3**. Temporal evolution of THz emission from LPE $WS_2$/InP with continuous irradiation of femtosecond IR pulses at a fluence of $4.42\times10^{-7}$ J/cm$^2$ at different conditions: (a) ambient conditions, pre-exposed sample, (b) vacuum ($1\times10^{-3}$ Pa) overnight, and then exposed in air, and (c) same measurement in (b) but with sample annealed at 400 K for 1.5 hours in vacuum.

Another advantage of TPTEM is the possibility of probing the local adsorption/desorption dynamics by decreasing the laser beam spot diameter during photo excitation. In our current experiment, we used a larger spot size of ~1.35 mm and a lower laser power of 30 mW to minimize the desorption of adsorbates due to irradiation with femtosecond laser pulses. To further minimize this effect, the femtosecond laser is blocked after every measurement. Figures 3a and 3b (in main text) show the temperature dependence of THz radiation from CVD graphene/InP before and after annealing at 445 K for 1 hour, respectively. Figures S4a and S4b show the temperature dependence of THz emission from LPE graphene/InP and LPE $WS_2$/InP, respectively, while Fig. S4c and S4d show emissions after annealing the samples. The measurements were taken under continuous vacuum pumping with pressure maintained at ~$1\times10^{-2}$ Pa. By increasing the temperature of the samples, two effects take place: one is the removal of adsorbates from sample surface by thermal desorption, the other is the enhancement of the surface field in the substrate at higher temperatures.[7] Both effects lead to a relative enhancement (more positive) in the THz emission from substrate, and the first as shown here and in our previous results[1] while the latter has been observed for semi-insulating GaAs.[7] This explains the difference in the temperature dependence of THz emission from unannealed and annealed samples. In unannealed samples, THz emission increases due to both effects, while in the annealed samples the increase in THz emission is due only to the enhancement of the surface field. Figures S4e and S4f show the $E_{unannealed} - E_{annealed} \cong E_{O_2}$ waveforms for LPE graphene/InP and LPE $WS_2$/InP, respectively.

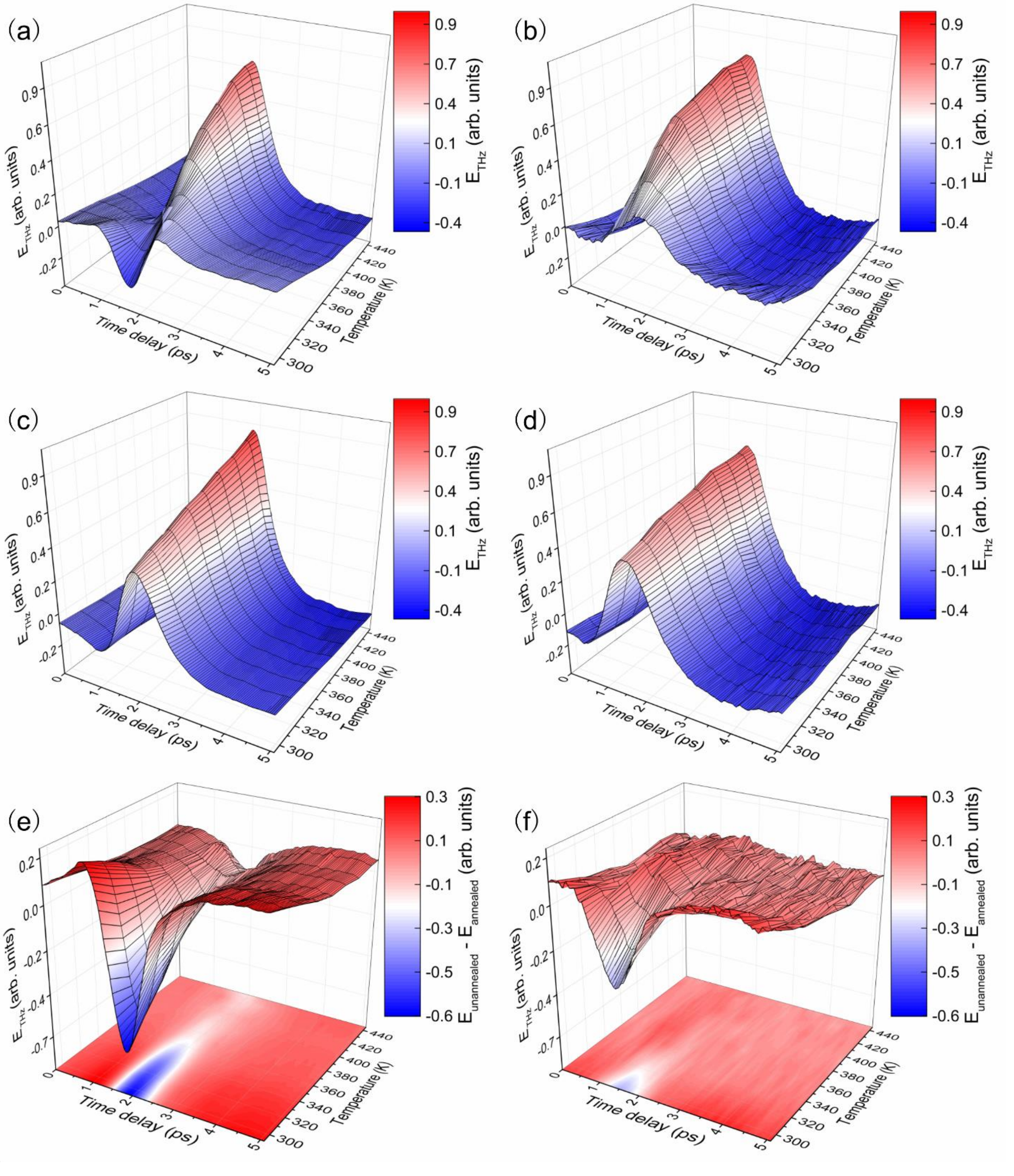


**Figure S4**. Temperature-dependence of THz emission from (a) LPE graphene/InP and (b) LPE $WS_2$/InP for sample pre-exposed in air. THz emission after annealing at 445 K for 1 hour in vacuum for (c) LPE graphene/InP and (d) LPE $WS_2$/InP. $E_{unannealed} - E_{annealed} \cong E_{O_2}$ at different temperatures for (e) LPE graphene/InP and (f) LPE $WS_2$/InP.

We used terahertz – time domain spectroscopy (THz-TDS) to measure changes in the optical conductivity (at 0.78 THz) of monolayer graphene due to removal of $O_2$ molecules from its surface upon annealing under vacuum conditions (Fig. S5). Adsorption of $O_2$ molecules is known to cause hole-doping in graphene. In the first annealing, graphene optical conductivity changed significantly from 300 K to ~450 K but remained almost constant above 450 K. We again measured the optical conductivity after cooling down the sample to room temperature and found no further significant changes within experimental error. The data in Fig. S5 suggest that there is no further significant desorption of $O_2$ molecules from graphene surface after ~450 K.

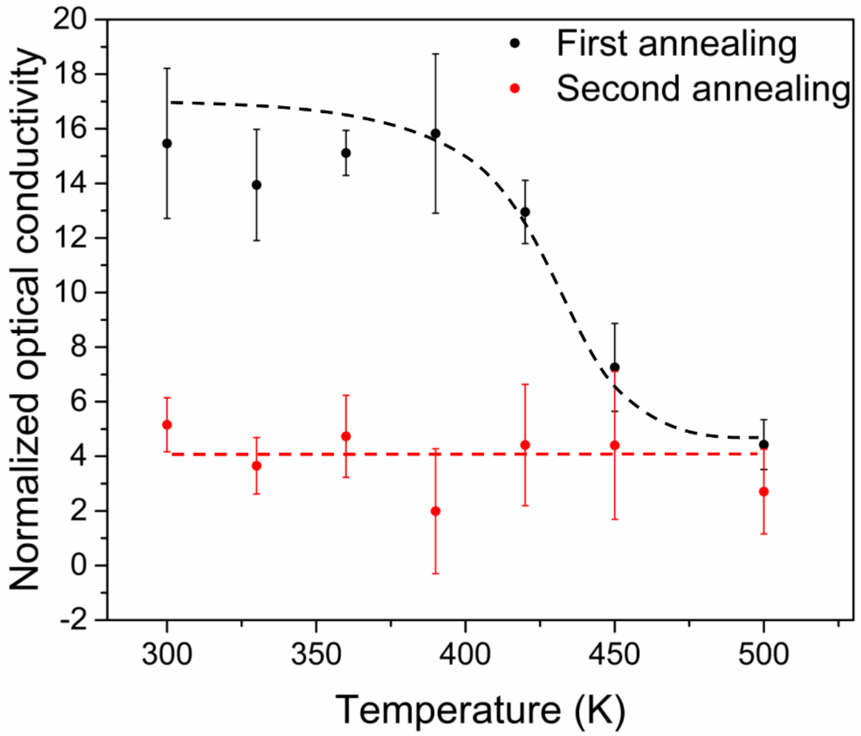


**Figure S5.** Temperature-dependence of normalized optical conductivity at 0.78 THz for monolayer graphene deposited on silicon substrate during first and second annealing. All measurements were taken under vacuum conditions. The dashed lines serve as guides for the eyes.

As discussed in the main text, the parameters $\mu(t)$, $n(t)$, and $E_d(t)$ given in equation (2) determine the observed THz field through equation (1). The carriers are generated well below the interface of graphene/InP since the penetration depth of 800-nm light in InP is 305 nm,[8] so the $\mu(t)$ in equation (2) is the electron/hole mobility for InP, which is not affected by the presence of $O_2$ in graphene. The optical absorption of graphene is affected by $O_2$ adsorbates,[9] which means that $n(t)$ might be dependent on the concentration of adsorbates in graphene since the IR excitation pulse is partially absorbed by the graphene sheet before generating carriers in InP. However, the fact that the current actually reverses direction means that this effect is less important than the effect of $O_2$ adsorbates to the $E_d(t)$ factor in equation (2). These arguments lead us to believe that during the $O_2$ adsorption/desorption process, the change in the THz radiation from graphene/InP is due to the change in $E_d(t)$, which in turn is caused by the electric field of the dipoles induced by the $O_2$ adsorbates in graphene.[1]

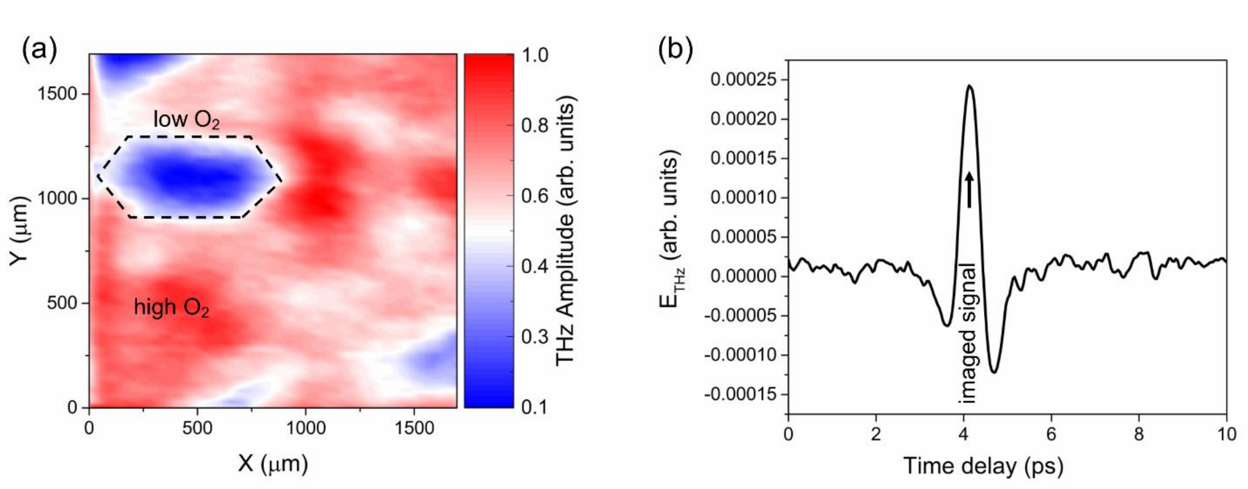


**Figure S6**. (a) THz amplitude mapping of CVD graphene/InP sample (pre-exposed in air) showing differences in the adsorbed $O_2$ molecules. The image in (a) was obtained by locking on the signal at ~4.2 ps as shown in (b) waveform of THz emission from sample.

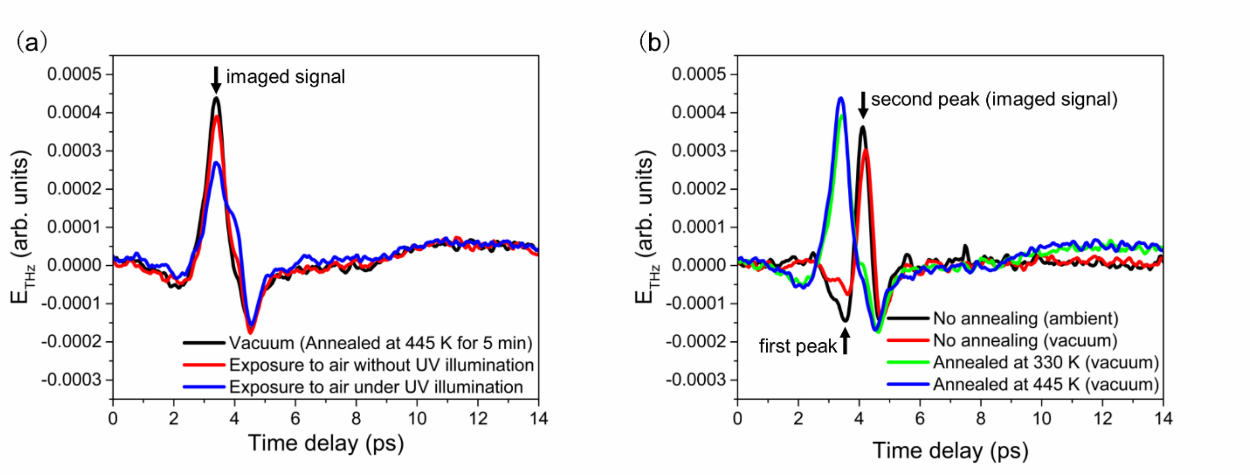


**Figure S7. Change in the waveforms of THz emission from CVD graphene/InP during the $O_2$ (a) adsorption and (b) desorption processes**. (a) THz emission from annealed CVD graphene/InP in vacuum showing waveform (black line) in the case where there are no $O_2$ adsorbates on its surface, and how the waveform changes upon $O_2$ adsorption by exposure to air (blue line) with and (red line) without UV illumination. (b) THz emission from CVD graphene/InP under ambient conditions showing waveform in the case where there are $O_2$ adsorbates in the surface of graphene (black line). Change in the THz emission from CVD graphene/InP due to the removal of $O_2$ adsorbates by (red line) vacuum pumping then by subsequent annealing at (green line) 330 K and at (blue line) 445 K under vacuum. All waveforms were obtained at room temperature and the THz amplitude mappings were obtained by locking into the signal as indicated in each figure.

To monitor the desorption process, first we exposed the sample in air, allowing enough $O_2$ to be adsorbed in graphene. We then took THz amplitude maps during the annealing process under vacuum conditions. The sample was cooled down to room temperature before each measurement since the emission from InP itself is temperature-dependent. THz amplitude maps were taken while the delay stage was fixed at the ~4.2 ps peak (Fig. S7b), and the measure of the desorbed $O_2$ was taken as the difference between THz amplitude mapping before and after annealing. The THz images taken under vacuum conditions (Figure S8a) before annealing, and the subsequent annealing (Figure S8b) at 330 K and (Figure S8c) at 445 K, show consistent decrease in the THz amplitude (at ~4.2 ps), which is indicative of $O_2$ desorption from the surface of the sample. Figure S8d is the spatial mapping of the relative amount of desorbed $O_2$ molecules during the annealing process from room temperature to 330 K, which shows significant $O_2$ removal with more than 50% desorption in the majority of the scanned area. The image also reveals a non-uniform $O_2$ desorption

from the surface, with some areas (inside the dashed enclosure) showing significantly higher desorption. From 330 K to 445 K, further desorption occurs except for the region inside the dashed circle in Figure S8e. This region in Figure S8e is the same region (region 5) in Fig. 5d in the main text, with the highest "natural" affinity to $O_2$ molecules. Spatial mapping of desorbed $O_2$ molecules during the entire annealing process from room temperature to 445 K (Figure S8f) shows almost complete desorption at this stage. Also, as previously mentioned, the identified areas in Fig. 5d showed different behaviors during the desorption process. This can be clearly seen by comparing the THz amplitudes of the indicated areas at each temperature, and we see that in the region identified as having high "natural" affinity to $O_2$ molecules (region 5), it seems that almost all adsorbates are gone after annealing at 330 K (Fig. S9a), whereas significant desorption of adsorbed $O_2$ is still evident in other regions during this stage of annealing (Fig. S9b and S9c). This result indicates that although more $O_2$ are adsorbed in region 5 (Fig. 5d in the main text), these are weakly bonded as they can be almost completely removed at a lower temperature. This observation can be roughly explained if one imagines the multilayer adsorption model (BET theory) wherein the second and the succeeding layers of adsorbates are attached to the adsorbent with a lower adsorption energy compared to the first layer.[10] In our results, the large initial $O_2$ adsorbate concentration in region 5 and the large amount of desorbed molecules after annealing at 330 K means a bigger change in the observed THz signal as seen in Fig. S9a.

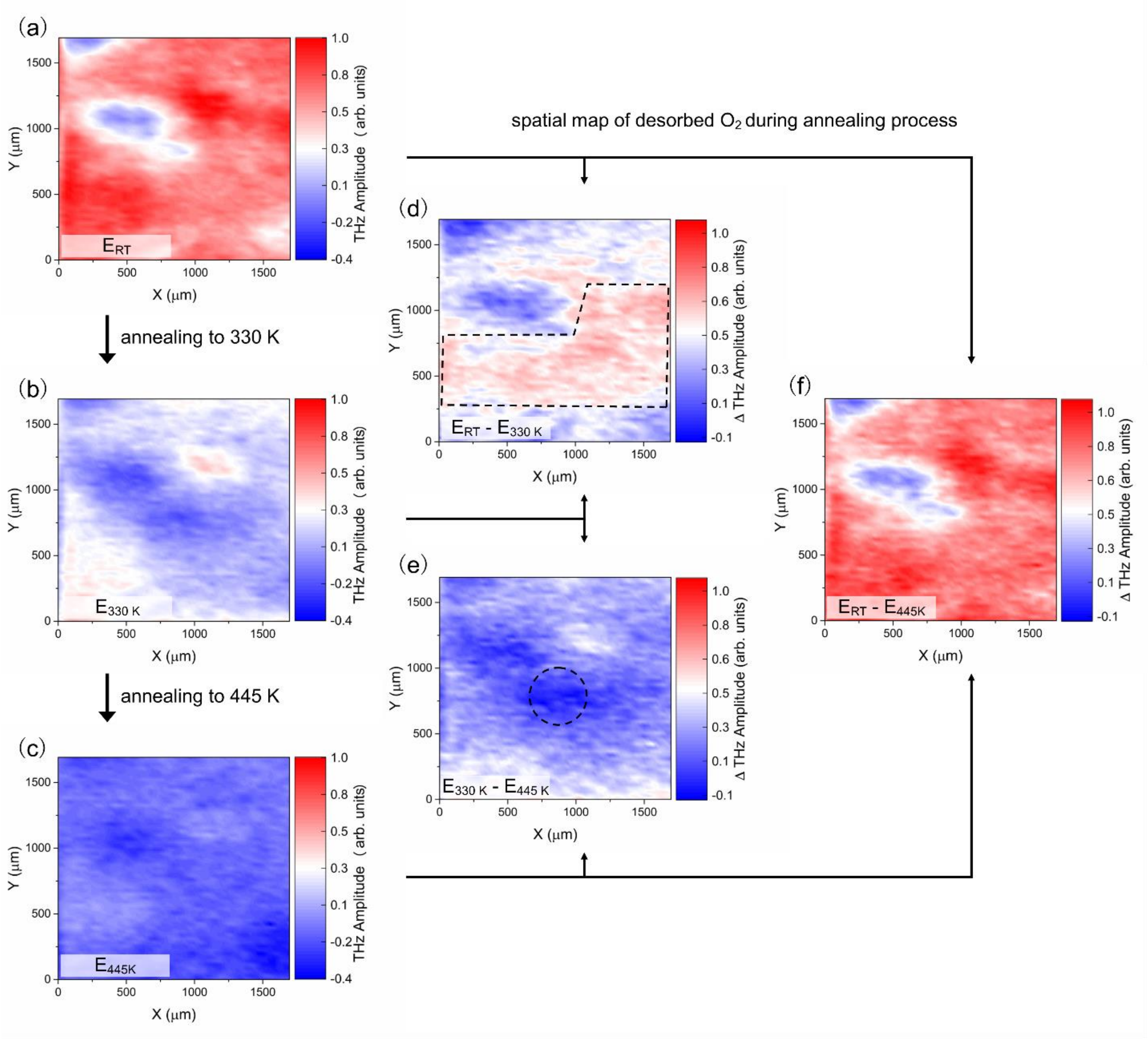


**Figure S8. THz amplitude mapping during $O_2$ desorption process by thermal annealing.** Amplitude mapping of THz emission from CVD graphene/InP (pre-exposed in air) taken under vacuum conditions (a) before annealing, and after annealing (b) at 330 K and (c) at 445 K. These images show consistent decrease of THz amplitude which is indicative of $O_2$ desorption from the sample surface. The difference in the THz mapping images before and after annealing gives a visualization of the desorbed $O_2$ molecules from the graphene surface. Spatial map of desorbed $O_2$ molecules during the annealing process from (d) room temperature to 330 K, (e) from 330 K to 445 K, and (f) the entire process from room temperature to 445 K. The image in (d) shows more than 50% $O_2$ desorption during this stage of the annealing process, and also reveals relatively higher desorption in the area inside the dashed enclosure. The region inside the dashed circle in (e), which shows almost complete desorption after annealing at 330 K, is the same region in Fig. 4d identified as a region with high "natural" affinity to $O_2$ molecules. Almost

complete desorption occurs after annealing at 445 K as shown in (f). In the THz amplitude maps (a-c), the blue (red) end of the scale signifies less (more) $O_2$ molecules on the graphene surface, while in the ΔTHz amplitude maps (d-f), the blue (red) end of the scale signifies less (more) $O_2$ molecules desorbed/removed from graphene during annealing.

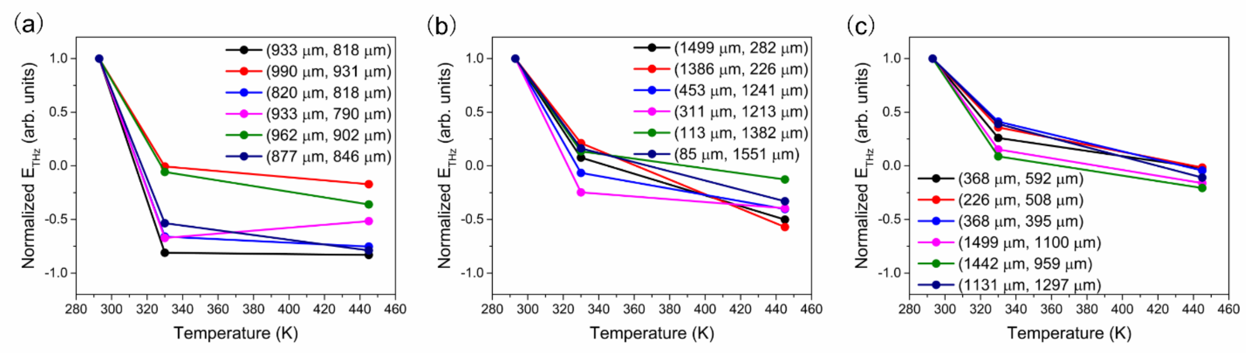


**Figure S9**. Normalized THz signal (at ~4.2-ps peak) from representative points in the identified regions in Fig. 4d at each stage of the annealing process: (a) high (region 5), (b) low (regions 1 and 2), and (c) fair/normal (regions 3 and 4) "natural" affinity to $O_2$ molecules. The decrease in the THz signal is correlated to the desorption of $O_2$ molecules. Annealing at 330 K results to almost complete desorption of $O_2$ molecules from region 5 as indicated in (a), which suggests weaker attachment of molecules in this area.

## References


1. Sano, Y. *et al.* Imaging molecular adsorption and desorption dynamics on graphene using terahertz emission spectroscopy. *Sci. Rep.* **4,** 6046 (2014).
2. Bagsican, F. R. *et al.* Laser THz emission spectroscopy of gas adsorption-desorption dynamics in tungsten disulfide nanosheets. *Surf. Sci. Nanotech* **14,** 78–82 (2016).
3. Lee, G. *et al.* Defect-engineered graphene chemical sensors with ultrahigh sensitivity. *Phys. Chem. Chem. Phys.* **18,** 14198–14204 (2016).
4. Zhang, Y.-H. *et al.* Understanding dopant and defect effect on $H_2S$ sensing performances of graphene: a first-principles study. *Comput. Mater. Sci.* **69,** 222–228 (2013).
5. Zhang, Y.-H. *et al.* Improving gas sensing properties of graphene by introducing dopants

and defects: a first-principles study. *Nanotechnology* **20,** 185504 (2009).
6. Liu, X.-Y., Zhang, J.-M., Xu, K.-W. & Ji, V. Improving $SO_2$ gas sensing properties of graphene by introducing dopant and defect: a first-principles study. *Appl. Surf. Sci.* **313,** 405–410 (2014).
7. Nakajima, M., Takahashi, M. & Hangyo, M. Strong enhancement of THz radiation intensity from semi-insulating GaAs surfaces at high temperatures. *Appl. Phys. Lett.* **81,** 1462 (2002).
8. Gu, P. & Tani, M. in *Terahertz Optoelectronics* (ed. Sakai, K.) 63–98 (Springer-Verlag Berlin Heidelberg, 2005). doi:10.1007/10828028_3
9. Santoso, I. *et al.* Tunable optical absorption and interactions in graphene via oxygen plasma. *Phys. Rev. B* **89,** 75134 (2014).
10. Brunauer, S., Emmett, P. H. & Teller, E. Adsorption of gases in multimolecular layers. *J. Am. Chem. Soc.* **60,** 309–319 (1938).
